# Long range physical cell-to-cell signalling via mitochondria inside membrane nanotubes: a hypothesis

**DOI:** 10.1186/s12976-016-0042-5

**Published:** 2016-06-06

**Authors:** Felix Scholkmann

**Affiliations:** Biomedical Optics Research Laboratory, Department of Neonatology, University Hospital Zurich, University of Zurich, Frauenklinikstr. 10, 8091 Zurich, Switzerland; Research Office for Complex Physical and Biological Systems (ROCoS), Mutschellenstr. 179, 8038 Zurich, Switzerland

**Keywords:** Membrane nanotubes, Mitochondrial networks, Mitochondrial reticulum, Filamentous mitochondria, Mitochondrial membrane potential, Ultra-weak photon emission, Cell-to-cell signalling, Long-range signalling

## Abstract

Coordinated interaction of single cells by cell-to-cell communication (signalling) enables complex behaviour necessary for the functioning of multicellular organisms. A quite newly discovered cell-to-cell signalling mechanism relies on nanotubular cell-co-cell connections, termed “membrane nanotubes” (MNTs). The present paper presents the hypothesis that mitochondria inside MNTs can form a connected structure (mitochondrial network) which enables the exchange of energy and signals between cells. It is proposed that two modes of energy and signal transmission may occur: electrical/electrochemical and electromagnetic (optical). Experimental work supporting the hypothesis is reviewed, and suggestions for future research regarding the discussed topic are given.

## Background

Cell-to-cell communication (signalling) is a crucial prerequisite for multicellular organisms that enables the emergence of complex behaviour evoked by the coordinated interaction of the single cells. Due to its fundamental function, a great variety of different cell-to-cell signalling mechanisms exist in parallel [1–4]. One quite newly discovered mechanism relies on nanotubular cell-to-cell connections, termed “membrane nanotubes” (MNTs), discovered just over 10 years ago [5]. Further research showed a great variety of functions of these MNTs including the exchange of different chemical and biological material [6, 7] as well as facilitation of electrical long-range coupling [8, 9]. There is accumulating evidence that mitochondria can be inside MNTs, possibly forming a connected structure (e.g., [10, 11]).

In the present paper, I review theoretical and experimental work that supports the hypothesis that mitochondria inside MNTs enable a long-range energy and signal exchange between cells. How to test this hypothesis, and what biological significance the hypothesis would have if correct, are also discussed.

## Novel findings regarding membrane nanotubes and mitochondria

### Direct cell-to-cell connections by membrane nanotubes

In 2004, the research group of H.-H. Gerdes published in a seminal paper in *Science* [5] compelling evidence of a “novel biological principle of cell-to-cell interaction” based on newly discovered “nanotubular structures”, termed “tunneling nanotubes”. The group was able to show that these nanotubular connections were present between different types of cells (rat pheochromocytoma (PC12), human embryonic kidney, and normal rat kidney cells), with diameters in the range of 50–200 nm and generally longer than single cells. MNTs can reach a length up to 1 mm as recently shown in human laryngeal squamous cell carcinoma cells [10].

Further research discovered that these “tunneling nanotubes”, later also called “membrane nanotubes” (MNTs) (the term also used in this paper), exhibit a large morphological and structural variety, despite the fact that all MNTs are filled with cytoplasm and have a lipid bilayer [12]. For example, most MNTs contain F-actin and some contain microtubules in addition (e.g., MNTs between primary neurons and astrocytes [13]). Önfelt et al. [14] discovered that all MNTs between human monocyte-derived macrophages contain F-actin, but microtubules were only present in thicker MNTs (i.e., having a diameter of > ~0.7 μm), indicating that the structural composition of MNTs not only depend on the specific cell type but also on the morphological features of the MNTs themselves.

The first detection of MNTs in vivo was published a few years later [15]. The analysis of MNTs in vivo showed that MNTs in this complex environment exhibit additional features not observed in previous studies; for example, MNTs in vivo can exist as contorted structures [16, 17], but MNTs forming straight tubes were also observed [18]. That individual MNTs could potentially stick together to form a single, thicker, MNT was reported recently [12].

Concerning the biological function of MNTs it was demonstrated that MNTs facilitate a great variety of different cell-to-cell communication mechanisms, ranging from the exchange of diverse signalling carriers (e.g., ions, proteins), organelles, bacteria, viruses [7, 19–22], or the spread of depolarisation which enables long-distance electrical coupling between cells [8, 9]. MNTs are good electrical conductors [8] with a conductivity in general larger than gap junctions [23].

MNTs play an important role in intercellular signal transduction in general, functioning of the immune system, micro- and nano-particle delivery processes, embryogenesis and development, differentiation and cellular reprogramming, apoptosis, cellular metabolic adaptation to stressors, cancer initiation and progression, and pathogen transfer, as recently reviewed by Sisakhtnezhad and Khosravi [21]. In addition, MNTs may have an electrophysiological function in neurobiological processes [24].

### Mitochondria inside membrane nanotubes

Several works observed that the cytoplasm inside MNTs can be (densely) occupied by mitochondria. This was shown for MNTs between liver macrophages [14], cardiomyocytes and cardiofibroblasts [25], endothelial cells and cancer cells [26], multipotent mesenchymal stem cells and vascular smooth muscle cells [27], mesenchymal stem cells and endothelial cells [11], cardiomyocytes and endothelial stem cells [28], human embryonic kidney cells and neuroblasoma cells [29], neural stem cells and brain microvascular endothelial cells [30], mesenchymal stem cells and cardiomyoblasts [31], bone-marrow-derived stromal cells and alveolar epithelial cells [32], rat pheochromocytoma cells [33], human peritonealmesothelial cells [34], primary human proximal tubular epithelial cells [35], human laryngeal squamous cell carcinoma cells [10], breast carcinoma cells [36], and rat hippocampal astrocytes [37].

The mitochondria inside MNTs can move, enabling a mitochondrial transfer between cells. Recently it was demonstrated that those MNTs that facilitate a mitochondrial transfer are also those that contain microtubules, and that the mitochondrial exchange can be regarded as a survival mechanism of damaged cells [33].

**Fig. 1:**
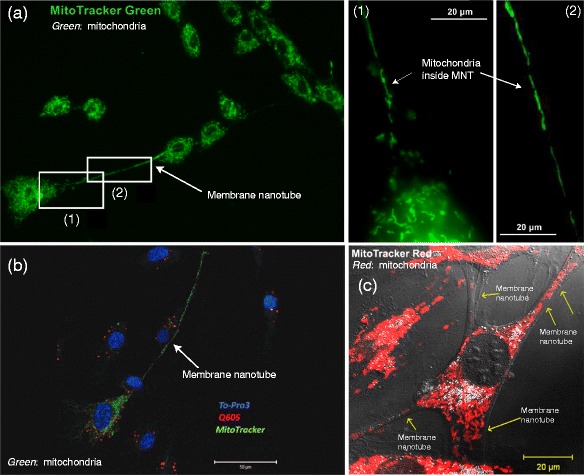
Examples for microscopic images showing mitochondria inside MNTs. **a** MNT between human laryngeal squamous carcinoma cells. Image modified from Antanavičiūtė et al. [10]. **b** MNT between human proximal tubular epithelial cells. Image modified from Domhan et al. [35]. **c** MNTs between cardiomyoblasts and mesenchymal stem cells. Image modified from Cselenyák et al. [31]

Importantly, the studies published so far about mitochondria inside MNTs not only showed that single mitochondria can be transferred between cells but that the density of mitochondria inside a MNT can be that high that the whole MNT seems to be filled with mitochondria (see Fig. 1). Antanavičiūtė et al. [10] highlighted this phenomenon by stating that a “dense network of mitochondria” could be observed in MNTs.

### Mitochondria: organelles with dynamic and diverse morphology

The outdated notion that mitochondria are generally spherical/ellipsoid organelles (having generally a diameter of 0.5–1 μm and a length of 1 μm) is increasingly being replaced by the modern view that mitochondria exist in a great variety of different shapes, and that their morphology also exhibits dynamical changes – a view now also adopted by established textbooks (e.g., [38]). The outdated view was basically due to the imaging of mitochondria by electron microscopy which lends itself to showing spherical/ellipsoid mitochondria. The reasons for this lies in the changes of mitochondria due to the sample preparation procedure (chemical fixation, dehydration and staining employed) and in the slice-based imaging that favours showing transverse sections of mitochondria [39].

Modern super-resolution microscopy made it possible to demonstrate that mitochondria exist in different forms ranging from spherical/ellipsoid shapes to long filamentous or tubular structures that can form also branched networks (reticula) of mitochondria [38]. In addition, the structure of the inner mitochondrial membrane (cristae) shows also different morphologies (e.g., disc-like lamellar, tubular, helical) [40, 41].

The variety of mitochondrial morphology can be described in approximation on a linear scale ranging from “fragmented” to “static hyperfused” (with the stated “microfused” and “dynamic hyperfused” in between) [42], or in a two-dimensional coordination system with the two axes “tubular/non-tubular” and “branched/condensed” [43], as well as in a four dimensional coordination system with the categories “size” (“smaller network” vs. “larger network”), “dynamics” (“over fused” vs. “over-fragmented”), “position” (“uniform distribution” vs. “asymmetric distribution”), and “shape” (from “non-tubulur” to “swollen tubes” to “less branching”) [44]. The morphology is dynamic and is determined by the ratio between mitochondrial fusion and fission [45–47], mitochondrial tubulation mediated by the member of the Kinesin 1 family KIF5B [48], movement along and attachment at the microtubule component of the cytoskeleton by dynein and kinesin motor proteins [49, 50] or without motor proteins [51], and attachment and interaction with actin filaments as part of the cytoskeleton [51, 52] as well as with the endoplasmatic reticulum [53–56]. According to recent theoretical work, the morphology of the mitochondrial network/reticulum is mainly determined by the “balance between anterograde and retrograde motility of mitochondria on microtubules” and by fusion/fission [57].

The morphological state of the mitochondria and their interaction forming mitochondrial networks is also strongly determined by the metabolic state. For example, inhibiting oxidative phosphorylation (OXPHOS) (by blocking the activity of complexes I and III) induces fission of the mitochondrial reticulum (“thread-grain transition”) (whereas inhibiting the F_0_F_1_-ATP synthase is not associated with fission) [58], and quiescent cells exhibit generally distributed mitochondria whereas dividing/differentiating cells show more mitochondrial networks [59, 60]. The morphology depends also on the phase of the cell cycle (G1: fused mitochondria forming interconnected networks, G1/S transition: large tubular networks, S: hyperfused giant mitochondria, M: fragmentation, i.e.,fissioned mitochondria) [61] and is linked to the mitochondrial membrane potential (Δψ_m_) [62–64] although the relationship is complex and the two variables can be also uncoupled [43, 65].

Additionally, the mitochondrial morphology depends on their spatial position inside the cell. Mitochondria naturally tend to aggregate around the nucleus (i.e., in the perinuclear region) and exist there in forms of mitochondrial networks; they also have a lower Δψ_m_ than the mitochondria in the periphery of the cell [58, 66]. A too large perinuclear agglomeration of mitochondria is indicative of cellular dysfunction, however [67]. The position of mitochondria in the cell is also determined to a large extend by the cytoskeleton, especially the microtubules radiating from the centrosome [68, 69].

It can be concluded that mitochondria inside a cell exhibit a great heterogeneity with respect to morphology and the metabolic state (indicated by Δψ_m_) [66, 70]; in this context, the term “mitodiversity” was recently coined [71].

### Mitochondria: sources of electrical currents and electromagnetic fields (non-radiating and radiating)

By applying novel voltage-sensitive nanoparticles Tyner et al. [72] could show that mitochondria are a strong source of intracellular electric (E) fields that can still be measured several μm away from the mitochondria in the cytosol (refuting the theoretical predictions that the field should be only significantly present at a distance of 1–10 nm beyond the mitochondrial membrane [73, 74]). The field strength is so strong that most probably the physiochemical properties of water around mitochondria are altered, as highlighted by Pollack [75] and Pokorný [76]. Also the water inside mitochondria is altered (higher viscosity [approx. 40 cP] [77], i.e.,15–20 times higher than the viscosity of the cytoplasm in erythrocytes [2.10–2.67 cP] [78], and more than 50 times higher than the viscosity of unbound “free” water [0.888 cP at 25 ° C; or 0.653 cP at 40 °C]) [79].

Concerning the finding of Tyner et al. of the extremely strong E-field around mitochondria it must be noted, however, that independent replications of it have not been published yet, and that the strong E-field around mitochondria are in contradiction with standard electrolyte theories predicting an E-field screening by mobile ions given by the Debye length. Further work is needed to clarify this issue.

The main source of the field is the mitochondrial membrane potential. Given a value of Δψ_m_ = −140 mV and a plasma membrane width of 5 nm, a field with a strength in the order of 3 × 10^7^ V/m is predicted (with sufficient agreement with the experimentally measured value of about 3 × 10^6^ V/m [72]). It is a quasi-static E-field, i.e., an E-field with slightly varying field strength depending on the continuous spontaneous variations of Δψ_m_ (“mitochondrial potential fluctuations” or “mitochondrial flickers”, as measured in various studies [80–85]). The fluctuations are generally in the very-low frequency range with period lengths in the order of seconds or minutes (e.g., see Figure 7 in [81], Figure 1 in [84], and Figure 3 in [82]). Concerning the amplitude of the Δψ_m_ fluctuations, O’Reilly et al. [85] measured a fluctuation amplitude of 17.6 ± 1.0 mV (range: 6–130 mV, skewed distribution: ~75 % of the values: < 20 mV) in a large population of mitochondria (*n* = 360). Interestingly, the amplitude was directly correlated to the resting Δψ_m_. The predicted E-field fluctuations are thus in the range of approx. 3 × 10^6^ V/m.

Charge movement along the respiratory chain causes another quasi-static field in mitochondria (having the largest field strength at complexes I, II and IV) which seems to be the driving factor for the proton translocation in the mitochondrial membrane due to the Lorentz force caused [86].

Despite these low-frequency non-radiation quasi-static fields (or “electromagnetic” fields according to the terminology that unifies electric and magnetic components based on current electrodynamic theory) there is convincing experimental evidence that mitochondria are also the source of radiating high-frequency electromagnetic fields in the optical spectral region, measured as spontaneous (low-level) chemiluminescence or ultra-weak photon emission (UPE). Pioneering work with this respect was already done in the 1960s by Stauff and Ostrowski [87] who detected spontaneous UPE (with a photomultiplier with a maximal spectral sensitivity around 400–600 nm) from rat liver mitochondria, the intensity of which was dependent on O_2_ supply, age of the mitochondrial suspension and temperature (decreasing the temperature increased the UPE). Reactions with reactive oxygen species (ROS) were assumed to be the cause. Mitochondrial respiration produces continuously superoxide (O_2_^•−^) in the mitochondrial matrix, predominantly on complex I, which can form hydrogen peroxide (H_2_O_2_) in subsequent steps (2 H^+^ + O_2_^•−^ + O_2_^•−^ → H_2_O_2_ + O_2_) [88]. The amount of O_2_^•−^ generated depends thereby on four main factors: the electrochemical transmembrane H^+^ potential difference Δp (with its constituents the transmembrane electrical potential Δψ and the H^+^ concentration difference ΔpH), the NADH/NAD^+^ and CoQH_2_/CoQ ratios, as well as the local O_2_ concentration. Superoxide (i.e., in its anionic form (superoxide anion radical, O_2_^•−^) and in its protonated form (HO_2_^•^) is constantly produced from complexes I and III of the electron transport chain due to a premature “leak” of oxygen during the energy transduction [89, 90]. It is often mentioned in the literature that 1–2 % of the O_2_ consumed by the mitochondria is released as ROS, however, this number only applies to the in vitro situation with altered states of the mitochondria and different environmental factors. The in vivo production rate is yet unknown but estimated to be “far, far lower” than 1–2 %, approx. 5–10 times lower, i.e., 0.1–0.4 % [88].

Despite the ROS-based generation of UPE, proton flows through cytochrome oxidase enzymes in the mitochondrial membrane by itself were predicted to be another source of UPE (in the near-infrared spectral region with a peak at approx. 900 nm) [91] – the experimental detection of this peak in the UPE spectrum is however not reported yet due to low-sensitivity of photodetectors in this spectral range.

Spontaneous UPE of mitochondria in the optical spectral range (from ultraviolet to infrared) could be detected however by several other groups [92–94], and it has been demonstrated that the UPE emission from mitochondria can be enhanced by the addition of iron [95–97], acetaldehyde [98], H_2_O_2_ or tert-butyl hydroperoxide [93], succinate [94], NADH [94], or a cocktail of doxorubicin and iron(III) chloride [99]. That mitochondria emit UPE even in the ultraviolet range was demonstrated by Zhuralev et al. [92] measuring UPE from isolated liver mitochondria of rats using a photomultiplier sensitive in the spectral range of approx. 320–620 nm. Ultraviolet UPE was also reported by Konev et al. [100] using a photomultiplier with sensitivity in the range of approx. 300–450 nm to measure UPE from yeast cells. Also measuring yeast cells, Quickenden and Hee reported ultraviolet UPE (even as low as 200 nm) [101].

Concerning the cause of mitochondrial UPE emission, Hideg et al. [94] observed that the broad emission spectrum (450–800 nm) resembles the chemiluminescent spectrum from a linoleic acid/lipoxygenase reaction. This, in addition with the fact that the mitochondrial UPE emission is quenched by the addition of cyanide and antimycin-A (disturbing the respiratory electron transport), leads to the conclusion that the emission is mainly due to excited carbonyls formed by lipid peroxidation due to singlet oxygen [94]. Cytochrome-*c* could also be important in these processes in form of a catalyst [94]. Concerning the origin of the ultraviolet UPE of mitochondria, Konev [102] concluded that tryptophan seems to be mainly responsible.

### Mitochondria as electrical transmission fibers

Already in the late 1960s, Skulachev from the Moscow State University came up with the conclusion that the electrochemical transmembrane H^+^ potential difference Δp (i.e., Δψ and ΔpH) can travel on a membrane so that membranes can be regarded as electric power-transmitting cables [103, 104]. Subsequent work supported that notion by reviewing evidence that Δp can be transported along membranes of different species/systems (i.e., demonstrated on Halobacterium membranes, chlorophyll-containing membranes, and cyanobacterial tichomes), and especially along the mitochondrial membrane [105]. Δp in the mitochondria is mainly formed by Δψ_m_, i.e., at 37 °C Δp is given by Δp [mV] = Δψ_m_ – 60 ΔpH_m_, with typical values for mitochondria of Δψ_m_ = 150 and ΔpH_m_ = −0.5 units, leading to Δp = 180 mV [106].

At least three charge transmission processes could take place on and in the mitochondria: (i) transmission of Δp (i.e., Δψ_m_ and ΔpH_m_) in form of diffusion of mobile ions (e.g., K^+^, Cl^−^, Na^+^), (ii) lateral movement of H^+^ along the mitochondrial membrane surface (proton current) “via membrane-bound water molecules forming ice-like structure” (with an increased transmission rate since proton conductivity is higher in structured water, i.e., ice or bound water) and H^+^ movement inside the mitochondria, and (iii) by lateral and intermembrane electron (e^−^) transport (electrical current) [105]. Proton conduction along the membranes, i.e., the water/lipid interface, has been demonstrated experimentally by several works [107–110], and the view that water structuring is involved in the interfacial proton conductance has been strengthened by recent experiments (e.g., [110]). There could also be a charge and signal processing taking place on the mitochondrial membrane that propagates (with a high speed in the order of ~1 m/s) like a pulse (i.e., a propagating deformation of the membrane) with subsequent changes in the physiochemical state of the membrane, as proposed recently by Fichtl et al. [111].

Support for the view that mitochondria function as intracellular power-transmitting cables comes from various experimental works. A seminal work was published in 1988 [39] showing that local photodamage (induced by laser irradiation) of mitochondria of fibroblasts or cardiomyoctes causes a breakdown of Δψ_m_ over the whole length of the filamentous mitochondria (about 40 μm) and the mitochondrial network (in fibroblasts), as well as in the irradiated spherical/ellipsoid mitochondria and connected clusters of mitochondria (in cardiomyocytes). This results not only showed that “filamentous mitochondria and mitochondrial reticulum represent electrically united (cable) systems” and that there could be a “long-distance transmission of the mitochondrial electrical potential”, but also highlighted the fact that the electrical connectivity of mitochondria in a cell is a heterogeneous phenomenon: filamentous mitochondria are electrical conductors, mitochondrial networks can be electrically connected (either by forming a reticulum or due to intermitochondrial junctions) whereby there are also electrically isolated mitochondria [39, 112]. Intermitochondrial junctions, having a high electrical conductance, might play a regulatory role enabling or disabling the electrical conductance of end-to-end-joined mitochondria forming a mitochondrial network [112]. This regulatory factor would explain the fact that parts of the mitochondrial network can have a different Δψ_m_ [113, 114].

According to the work of De Giorgi et al. [115], changes of the gating status (i.e., opening and closing) of the mitochondrial permeability transition pore (PTP) causes changed Δp which can propagate along the network, i.e., mitochondria “can form a dynamical proton-conducting network capable to propagate and commute the electrical signals locally generated during PTP gatings”.

The degree of electrical coupling between mitochondria depends on many factors, as for example the redox state of the cytoplasm. Local photodamage of a mitochondrial reticulum in HeLa cells showed no large spread of depolarisation, indicating an only small size of electrically-connected mitochondria. However, cells pretreated with MitoQ (an antioxidant) before performing the local mitochondrial photodamage showed that “the area of depolarisation around local photodamage drastically increased and reached 30–50 % of total mitochondrial population” [58].

Recently, as published in *Science*, Glancy et al. [116] showed that the primary pathway of skeletal muscle energy distribution is “membrane potential conduction via the mitochondrial reticulum”, in agreement with the early work of Skulachev about mitochondria acting as electric power-transmitting cables [103–105].

**Fig. 2:**
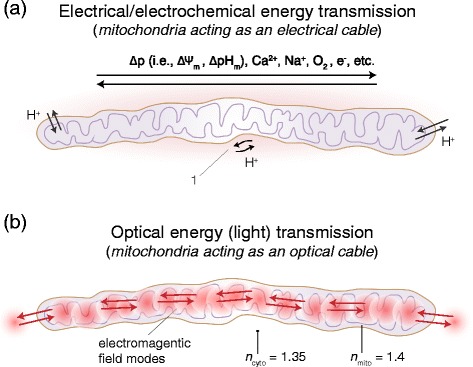
The two possible physical energy transmission mechanisms in mitochondria: **a** electrical/electrochemical, and **b** optical (*light*). The arrows indicate the possible transmission directions. 1: proton conductance along the mitochondrial membrane. Whereas the existence of mechanism **a** is experimentally proven, mechanism **b** is currently only predicted by theory and no experimental investigation into the correctness of the prediction has yet been published

A visualisation of the electrical transmission mechanism in mitochondria is shown in Fig. 2a.

### Spatio-temporal oscillations in Δψ_m_ of mitochondrial networks: an emergent property of mitochondrial coupling

If mitochondria inside a cell are generally not functionally isolated but can be linked together electrically/electrochemically, emergent network properties (like synchronized and wave-like spreading changes of Δψ_m_) should be observed. Indeed this is the case and was reported [117–119]. Individual mitochondria and whole networks show inter-mitochondrial coupling and synchronization due to “mitochondrion-to-mitochondrion coupling” [117]. The Δψ_m_ of the whole network can oscillate, especially if the redox state of the cell is shifted to more oxidized reactions. This phenomenon was shown in mitochondria of intact pig heart cardiac myocytes (mean Δψ_m_ frequency (*f*): *f* = 10.30–58.1 mHz, corresponding to a period length (*T*) of *T* ≈ 17–97 s). Interestingly, on the mitochondrial network of freshly isolated myocytes, the mean frequency of synchronized Δψ_m_ oscillations was found to depend on the type of medium; i.e., with glucose: *f* = 8.73–22.3 mHz (*T* = 44.8–114 s), with pyruvate: *f* = 3.7–54.83 mHz (*T* = 18–270 s), with lactate: *f* = 3.9–15.0 mHz (*T* = 66–256 s), with *β*-hydroxybutyrate: *f* = 4.0–10.1 mHz (*T* = 11–99 s). The mean frequency was found to be correlated with the mitochondrial cluster size displaying the synchronized oscillations: the lower *f*, the higher the synchronized mitochondrial cluster area. This “large-scale synchronization in mitochondrial dynamics” documented by Kurz et al. [117] could be a result of the physical and electrical coupling of mitochondria as described by Skulachev. In addition, wave-like ROS-induced ROS releases [120–123] among neighboring mitochondria seem to play a role too. Physiochemically connected mitochondria thus exhibit self-organized dynamic coupling leading to synchronized oscillations – a form of “mitochondrial criticality” as defined by Aon et al. [124, 125], and possibly also related to fluctuations of the mitochondrial redox state ([NAD(P)H/NAD(P)^+^]) [126] Δψ_m_ [127] showing temporal scale-invariant long-range correlations. Aon et al. concluded that “cardiac mitochondria behave as a network of coupled oscillators under both physiological and pathophysiological conditions” [127]. When the number of fluctuating mitochondria in a network exceeds a critical threshold value (i.e., a percolation threshold), a global phase transition of the network state occurs, leading to spontaneous synchronisation of the fluctuations so that a coherent oscillation emerges [127].

### Electrical mitochondrial coupling: biological significance for energy and signal transmission

Why do mitochondria form mitochondrial networks and exhibit electrical coupling? An answer to this question was recently given by a study conducted by Hoizing et al. [42] in which it was concluded that three main reasons can be given: (i) increased ATP production (as experimentally observed [128–130]), (ii) increased robustness against perturbations (e.g., fused mitochondria are more robust against stress than non-fused ones [131]), and (iii) mitochondrial quality control (functionally healthy mitochondria have a higher probability to fuse [132]). In addition, the hypothesis of Skulachev [105, 112] should be considered that mitochondrial networks or filamentous mitochondria enable energy transmission from parts of the cell with higher O_2_ concentration to parts with lower O_2_ concentration, enabling OXPHOS also in these regions. Coupled mitochondria might also even facilitate O_2_ transport along the inner mitochondrial membrane [39] and enable a fast energy transmission in general [116].

### Mitochondria as optical transmission fibers

In 2004 Thar and Kühl [133] published a paper in which they concluded, based on a review of experimental facts, that there could be “electromagnetic radiation propagating along the mitochondrial reticulum” due to the “light guiding properties of the mitochondrial network”, facilitating “long-range interaction between individual mitochondria”. The authors derived this conclusion by analyzing the biophysical properties of mitochondria and the cytoplasm: filamentous mitochondria resemble optical waveguides with a core refractive index (*n*) higher than the index of the cladding (*n*_mito_ = 1.4, *n*_cyto_ = 1.35) and a cutoff wavelength (*λ*_c_) of 145 nm (*λ*_c_ = 1.305 *D*(*n*_mito_^2^ – *n*_cyto_^2^)^1/2^, with *D* = 300 nm the average thickness of a filamentous mitochondrion), i.e., mitochondria could act like single-mode optical fibers (waveguides) enabling the transport of electromagnetic radiation with a wavelength higher than 145 nm. That mitochondria are indeed a source of optical radiation (in the wavelength rang of approx. 450–750 nm) was shown in “Mitochondria: sources of electrical currents and electromagnetic fields (non-radiating and radiating)”. Thar and Kühl, being aware of the experimental work about the mitochondrial spontaneous low-level chemiluminescence, hypothesized that the “light generated in one mitochondrion could propagate along the network and, e.g., trigger some chemical reaction in another mitochondrion.”

Thar and Kühl even postulated that the inner mitochondrial structure (with a refractive index of the matrix of *n* = 1.5 and of the intramembrane space of *n* = 1.35, as well as the lamellar cristae structure) resembles an optical multi-layer system possibly enabling a light amplification mechanism due to induced emission and optical feedback (comparable to a distributed feedback laser). An experimental proof of this hypothesis is lacking though due to current technical limitations of detecting the optical emission of single mitochondria and analyzing it for their coherence-properties. However, the simulation by Thar and Kühl of the reflectance and transmittance spectra of a model mitochondrion measuring 5 μm in length and showing the light amplification mechanism delivered spectra with maxima in the region of approx. 400–550 nm, close to the spectral peaks of the ultra-weak photon emission from isolated mitochondria [94].

The optical radiation could also not only be transmitted in directly fused or connected mitochondria but also between mitochondria separated with a gap when the distance of the gap is smaller than the optical wavelength [133]. The observation that mitochondria can regulate their activity even when separated with a quartz glass [134] might be a related phenomenon.

The optical electromagnetic radiation may also couple into microtubules attached to the mitochondria (due to the evanescence field around the mitochondria), or vice versa [133]. Microtubules (having a refractive index of *n*_microt_ = 1.51 [135]) can also be regarded as electromagnetic (optical) wave-guides (with a cutoff wavelength of approx. 21 nm, using a diameter value of the microtubules of 24 nm [136]). However, the cutoff wavelength is in the extreme ultraviolet spectral range and such radiation would automatically create large damages of the microtubules due to the ionizing effect of this kind of highly energetically radiation. Thus, an optical near-field coupling between mitochondria and microtubules seems to be not likely.

However, there could be an optical near-field coupling between mitochondria and the cellular nucleus due to refractive-index matching (the refractive index of the nucleus, *n* = 1.355–1.365 [137], is similar to the mitochondrial one), forming an electromagnetically energy (and possibly also signal) transmission system. Such a scenario is interesting to consider since it is known that there is a clustering of mitochondria around the nucleus in the cell [138–143], and even direct attachment of mitochondria to the nucleus (e.g., [144]).

But is the flux (photons/time) of UPE sufficient to enable such an optical-based energy and signal transmission in general? Given the fact the production rate of ROS by mitochondria is quite low 0.1–0.4 % and that the amount of photons produced by the excited species is very low too (much lower than 10^−5^ [145]), it seems to be unlikely that that amount of UPE flux can have any regulative meaning for biological reactions. However, when assuming that there are additional sources of UPE generation in mitochondria (as summarized) and that mitochondria may enable light-amplification as proposed by Thar and Kühl the possibility of much higher UPE fluxes in mitochondria should not be ruled out. Also the measured UPE flux may not represent the in vivo UPE flux *per se* since only a fraction of the UPE produced by mitochondria may be emitted into the cytoplasm. The function of mitochondria as optical cables would prevent the escape of UPE from the optical medium which enables the light guiding effect, i.e., the inner space of mitochondria with the mitochondrial membrane as the “cladding” of the “optical fiber”.

For a visualisation of the hypothesized optical transmission in mitochondria please refer to Fig. 2b.

### Optical mitochondrial coupling: biological significance for energy and signal transmission

Concerning the function of mitochondrial electromagnetic (optical) energy and signal transmission in the biological context, a few suggestions have been published so far.

The most recent was published by Bagkos et al. [146], putting forward the idea that the optical emission of mitochondria might be a factor involved in mitochondrial retrograde signalling (i.e., the regulating of nuclear gene expression by mitochondrial signals [147–149]). Bagkos et al. propose the following cascade of events happening in the cell: (i) mitochondria cause UPE, (ii) light-sensitive nuclear receptors in the nuclear membrane absorb the light, and (iii) the activation regulates nuclear gene expression. Since each nuclear receptor has a specific absorption spectrum (which peaks generally in the optical region, i.e., visible and near-infrared), changes in the spectral composition of UPE lead to the activation of specific nuclear receptors, deepening on the match between the emission and absorption spectra.

Work by Albrecht-Buehler showed that cells form surface projections to infrared light sources, but only when the light was pulsed (e.g., 1 pulse/s) [150]. Later experiments revealed that the centrosome of the cell seemingly functions as a photoreceptor [151]. If endogenous UPE can activate the centrosome in the same way is currently unknown, however. Centrosomes are important structures in the cells, not only because of their role as microtubule organizer but also since they function as “coordination centres in eukaryotic cells, at which specific cytoplasmic proteins interact at high concentrations and important cell decisions are made” [152]. The significance of centrosomes is also highlighted by the fact that abnormalities (concerning structure, size, number) are a “hallmark” of cancer cells, and that this phenomeonon “may play a dominant role in tumour initiation and progression” [153]. In addition, synchronized electric oscillations of the centrosome, microtubules and chromosomes are a source of an quasi-static field that may play an important part in the regulation of mitosis and meiosis, according to the hypothesis of Zhao and Zhan [154].

Another mechanism how (weak) light could play a role in cellular physiology was already proposed in the 1980s by Kato et al. [155]: cytochrome *c* oxidase (COX), the terminal enzyme of the respiratory chain in mitochondria, acts like a photoreceptor. This reasoning was further strengthened by the work of T. Karu [156]: she discovered that the characteristics of response of cells to light (i.e., the photobiological response) vary according to the absorption spectrum of COX. Peaks in both the cell biological reactivity as well as the COX absorption spectrum are in the red to near-infrared optical region [156, 157]. In this spectral region, differences in the wavelength of the irradiated light can have opposite cellular effects, as observed for example by Gordon and Surry [158] (650 nm: increase in OXPHOS, 725 nm: decrease), and Karu et al. [159] (632.8 nm: increase in intracellular cyclic adenosine monophosphate (cAMP) concentration, 760 nm: decrease), as summarized by Karu et al. [156]. This behavior can be explained by taking into account that the redox state as well as specific single chromophores of COX also have different absorption maxima, i.e., the Cu_*A*_ site of COX absorbs maximally at 620 nm (reduced state) or 830 nm (oxidized state), and the Cu_*B*_ size at 760 nm (reduced) or 680 nm (oxidized) [156]. The light-mediated activation of COX is followed by a change in the redox state of the mitochondrion and thus eventually of the whole cell. It has not been investigated experimentally whether the endogenous UPE, generated and transmitted by mitochondria, is able to activate COX.

In addition, the impact of light on water properties must be mentioned. It was shown that the water layer on hydrophilic surfaces (which exist in a specific phase [160]) increased in size due to light exposure, especially in the infrared region [161]. The specific state of water (e.g., higher viscosity) inside mitochondria and around mitochondria could thus be also influenced by the UPE produced by mitochondria. Another effect, proposed recently by Sommer et al. [162], is that near-infrared light seems to influence the viscosity of water inside mitochondria. Near-infrared light exposure is linked to decreased water viscosity that could enhance the performance of the ATP synthase due to nanomechanical improvements of the rotational mechanism.

Moreover, there are theoretical works that assign UPE a functional role in the development and the functioning of the optical visual system [163–174].

Finally, there is a prediction, already made in the 1970s [175], that there could be a mechanism of gene regulation based on the following signal process: (i) UPE is generated by mitochondria, (ii) UPE interacts with the tryptophan in proteins attached to the deoxyribonucleic acid (DNA) polymers in the cell due to matching of the UPE spectra of mitochondria and absorption spectra of tryptophan (maximum in the UV range), (iii) the interaction leads to a change in the electrical conductance of the DNA, followed by (iv) further processes that finally lead to gene regulation. Unfortunately, no follow-up work considering this hypothesis has been published so far.

## Hypothesis: energy and signal exchange between cells via mitochondrial fibers inside MNTs

**Fig. 3:**
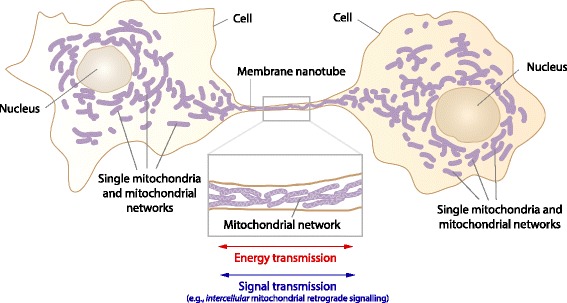
Visualization of the hypothesis that mitochondria inside MNTs enable energy and signal exchange between cells

Based on the information given in the previous sections, the following hypothesis is proposed: mitochondria inside MNTs enable energy and signal exchange between cells. Key aspects of the proposed hypothesis are visualized by Fig. 3. The hypothesis can be divided into three parts:

1. Mitochondria inside MNTs, formed between cells, can exist as a connected structure either (i) as filamentous mitochondria connected to each other via intermitochondrial junctions, (ii) in the form of a mitochondrial network, or (iii) as a combination of both forms.
2. The mitochondrial connection via MNTs functions as a structure conducting (i) charges (acting as an electrical cable), and/or (ii) electromagnetic radiation in the optical wavelength region (acting as an optical cable).
3. Both the electrical and optical energy transmitted by the mitochondria inside MNTs allow for (i) energy as well as (ii) signal exchange between cells.

How well is the above hypothesis supported by experimental evidence?

The predicted continuous/connected mitochondria inside MNTs (part 1 of the hypothesis) has not yet been explicitly investigated and proven. However, the existence of mitochondria inside MNTs is proven, and that the mitochondria form cable-like structures inside MNTs can be seen in several microscopic images published (see Fig. 1).

Concerning the predicted transfer of charges and electromagnetic radiation along mitochondria inside MNTs (part 2 of the hypothesis), no experiments have been performed. That there could be an electrical coupling between cells via MNTs has been demonstrated [8, 9, 13, 176]. However, the question of whether mitochondria can be involved in the electrical cell-to-cell coupling has not been addressed so far. Charge transport along filamentous mitochondria and networks is proven to occur and the notation that mitochondria act as “electrical transmission fibers” agrees with experimental findings (see “Mitochondria as electrical transmission fibers”). That mitochondria are also generating electromagnetic radiation in the optical wavelength range is proven (see “Mitochondria: sources of electrical currents and electromagnetic fields (non-radiating and radiating)”). The ability of mitochondria to transmit this radiation (or even amplify it) has not been demonstrated experimentally yet but was predicted theoretically (see “Mitochondria: sources of electrical currents and electromagnetic fields (non-radiating and radiating)”).

Part 3 of the hypothesis states that electrical and optical energy is potentially transmitted between the cells by the mitochondria inside MNTs to enable a transfer of energy and signals, i.e., the transmission facilitates physiological processes linked to cell-to-cell communication. That cell-to-cell coupling via MNTs occurs and that this coupling serves important physiological functions (e.g., during development) is proven [6, 21, 177–179]. That the functional cell-to-cell coupling can be due to the processes described in part 3 of the hypothesis has not been investigated so far. One scenario could be that these processes enable an “intercellular mitochondrial retrograde signalling”, i.e., the mitochondria in cell *A* could affect the nuclear gene expression of cell *B* (and vice versa) due to the physical coupling via mitochondria inside MNTs. Also the cell-to-cell coupling could enable an intercellular information exchange that couples the mitochondrial network of the cells together which could lead to novel emergent behaviour of the mitochondrial networks. Such emergent behaviour might even enable the storage of information or intracellular signal-processing, as recently suggested to happen in mitochondrial networks [180].

## Conclusions

In the present paper I reviewed the current knowledge about the possible role of mitochondria in MNTs for cell-to-cell communication based on physical energy and signal transmission. The proposed hypothesis (i.e., the three parts of it see “Hypothesis: Energy and signal exchange between cells via mitochondrial fibers inside MNTs”) should be tested by novel cell biological experiments.

**Fig. 4:**
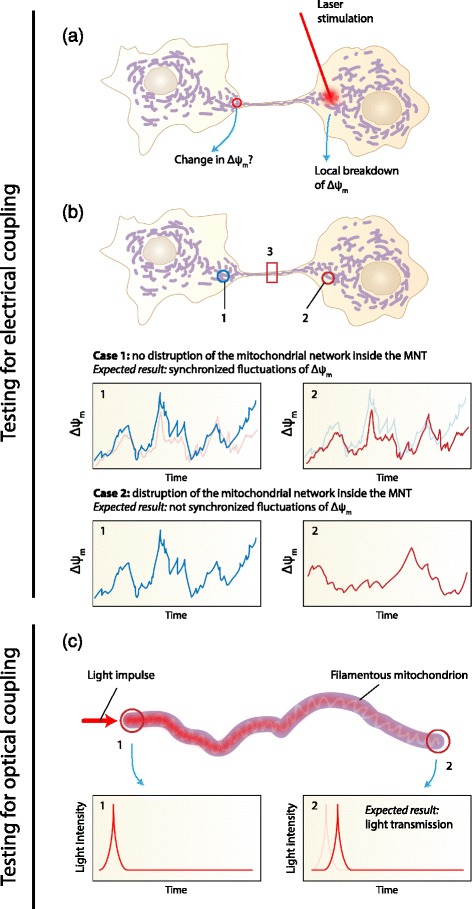
Visualization of the possible experiments to test the proposed hypothesis. **a** Laser stimulation to locally disrupt the Δψ_m_ in one cell. It is then tested if the mitochondrial network in the cell connected by the MNT also shows a breakdown of Δψ_m_. **b** Monitoring the spontaneous fluctuations of Δψ_m_ in both cells connected by a MNT filled with connected mitochondria. A synchronization of the fluctuations should be observed (case 1). When disrupting the mitochondrial connectivity (at point 3) of the mitochondrial network inside the MNT the synchronization should disappear (case 2). **c** Shining light into a filamentous mitochondrion and measuring the light intensity at the other end of it will proof if mitochondria can act as optical fibers

The proposed hypothesis may be tested by the following experiments (see also Fig. 4):

1. *Experiment 1* (testing for electrical coupling): The experiment should be performed on two cells (A and B) that are connected by a MNT. It must be ensured that the MNT is filled by a mitochondrial network. The electrophysiological state of it (Δψ_m_) is monitored by fluorescence probes. The mitochondrial network of cell A is then disturbed by shining a laser pulse on the part of the mitochondrial network present in cell A. If the hypothesis of an electrical coupling of cells via the mitochondria inside MNTs is true this local disruption of Δψ_m_ in cell A will travel along the mitochondrial network in the MNT so that also the part of the mitochondrial network present in cell B will be effected (i.e., it will show a disruption of Δψ_m_ as well). This experiment is similar to the experiment already done by Amchenkova et al. [39] investigating the electrical coupling within a mitochondrial network.
2. *Experiment 2* (testing for electrical coupling): As another possibility to test whether cells connected by a MNTs with mitochondria inside show electrical coupling is analysing the spontaneous fluctuations of Δψ_m_ in cell A and B. A synchronous fluctuation is indicative for an electrical coupling via the MNT connections (case 1). When disrupting the mitochondrial continuity inside the MTT, the synchronization should disappear (case 2).
3. *Experiment 3* (testing for optical coupling): A direct test for optical coupling of cells via a mitochondrial network inside a MNT is hard to realize due to the extremely weak light to be expected to be present and the necessity to measure it very locally (i.e., in the nm range). In addition, since it is expected that the optical radiation travels inside the mitochondrial network (i.e., shielded from the environment) any measurement aiming to measure the radiation is faced with the challenge that the measurement process will most probably affect the measurement results since the optical guidance properties of the mitochondrial network will be changed. However, the following experiment could be imagined: At one end of a mitochondrial network a laser pulse is applied and at the other end of it is tested if the light impulse can be detected. This would demonstrate the ability of a mitochondrial network of acting like a waveguide in the optical spectral region.

Furthermore, additional experimental (and theoretical) work is needed to investigate further related questions such as (i) whether the electrical and electromagnetic signals/radiation also couples to other cellular structures, as already proposed by several works [133, 181–187], and (ii) how this interaction between different networks (e.g., mitochondrial, cytoskeleton, endoplasmatic reticulum) might be important for inter- and intracellular signalling and organization.

Finally, it must be investigated what other possibilities for electrical coupling of cells via MNTs exist. Especially two aspects should be investigated in detail: coupling via *actin* and/or *microtubules*. F-actin is present in most MNTs, microtubules in some [13, 14]. Actin can transmit electrical signals [188–193], and microtubules have this property too [192–201]. It is thus also likely that there are different possibilities and routes of electrical signalling between cells via a MNT: by mitochondria, actin and/or microtubules. It is possible that all three pathways can be active in parallel so that a multi-modal electrical signalling could happen between cells connected by a MNT. In the present manuscript I focused on mitochondria; the possible involvement of actin and microtubules in MNT based electrical cell-to-cell coupling should be a topic for future research and work.
